# Therapeutics

**Published:** 1878-04

**Authors:** 


### Therapeutics.

Action of Cathartics. Dr. L. Brieger. (*Arch. f. Exp. Path., &c., VIII, 4 and* 5, 1878.)

Beginning with a recapitulation of the present state of the question, the author thereupon details his experiments made in Cohnheim's labaratory. Dogs were kept without food for 2 or 3 days, and then narcotized with morphine. Their small intestines were exposed, a certain length of them isolated by two ligatures; the piece included was washed by an injection of water and thereupon emptied completely. It was next divided into three equal parts by the application of two more ligatures. Into the two peripheral parts the drug was injected with a hypodermic syringe; the central portion was used for comparison. The gut was returned into its cavity and the animal killed after the lapse of 4½ hours.

### A. Saline Cathartics.

I. A one per cent. solution of sulphate of magnesium was absorbed after injection without reaction.

II. An injection of 5 grammes of a 20 per cent. solution of the same agent caused a distension of ligatured intestine with an alkaline fluid, containing mucous flocculi soluble in liquor sodæ. This fluid changed starch into sugar, and digested raw fibrine, but not boiled fibrine or albumen. Microscopically, it contained mucous corpuscles and epithelium. The mucous membrane was pale and normal.

III. Similar results were obtained from solutions of magnesic sulphate (50 per cent.), sodic sulphate (saturated sol.) and sodic chloride (saturated sol.) Two grammes ($\frac{1}{2}$ ʒ) of the latter gave rise to 13 grammes of exsudation.

B. DRASTICS.

Half a drop of croton oil (dissolved in 2,5 c. cm. ether) filled the gut with 18 grammes of sanguinolent fluid.

Ext. colocynth dissolved in two grm. water had the following effect:

0,02 grm. No exudation, mucous membrane reddened. 0,04, bloody fluid, inflammation. 0,05, distension with bloody fluid. 0,1, diphtheritic inflammation of the mucous membrane. These fluids possessed the same properties, as those produced by salines, and contained besides a large number of white and red corpuscles, epithelium, and flocculi of mucus and fibrine.

C. LAXATIVES.

Calomel, infus. sennæ, pulv. rhei, ext. aloes, ol. ricini, were injected in variable quantities. The gut, examined after four to seven hours, was found *simply* contracted, its surface pale. The water used as menstruum had been absorbed, the oil and the other agents remained unchanged.

The author deduces from these experiments, that salines act by causing both transudation into the intestine, and increased secretion of the intestinal glands (since the corresponding fluid possessed the physiological properties of intestinal juice). The effect of the laxatives of class C must be accounted for by their power of stimulating intestinal peristaltic movements, and hence hastening the passage of the ingesta before the fluid part can be absorbed. The drastics, on the other hand, may also stimulate peristalsis in weak doses, while in larger quantities they cause an inflammatory exudation and hypersecretion.—(*Allg. Med. Central Zeitung*, 1878. No. 10 and 11.)

THE USE OF HYDROBROMATE OF QUININE IN DISEASES OF CHILDREN. Dr. Steinitz of Breslau. (*Allg. Med. Central Zeitung.*)

The author has used the remedy in an extensively prevailing epidemic of hooping-cough, giving it generally in a mixture composed of three to five parts of the hydrobromate in one thousand of syrup, the dose being a teaspoonful every two hours. In no case was it necessary to use any other remedies. The hooping-cough had in twenty-three cases lasted on an average ten weeks, and in fifteen others twelve weeks ; and in the use of the remedy the paroxysms became in the course of a week less frequent and milder. No after-effects upon the alimentary canal were discovered. Three deaths occurred, all in very weak and scrofulous individuals, in whom other complications were present. Dr. Steinitz takes the opportunity of remarking that he prescribed in several cases the extract of castanea vesica, which has been extolled as a remedy, but without good results.

He also used the hydrobromate of quinine in cases of spasms of the glottis. Three of the patients died after only a few paroxysms. The remaining six recovered. The medicine was prescribed as stated above, and was borne well. In all the six cases the attacks diminished, at times varying from the third to the fifth week, in intensity as well as in frequency ; and the duration of the disease was in no case longer than from four to six months. This result is satisfactory when compared with the previous course of the disease under the use of other medicines, such as bromide of potassium, oxide of zinc, valerian, and musk, none of which could be borne for several months together.

Dr. Steinitz has also given the hydrobromate of quinine in the dental convulsions of children, but cannot as yet speak of its efficacy in this malady. He regards it, however, as deserving a trial.

A New Revulsive.—Conturier. (*L'Union Médicale*, 22d December, 1877.)

Among the usual medicines there are few which render as much service as revulsives. Sinapisms are of daily use, and flying blisters, although reserved for graver cases, have also numerous indications. But there are many circumstances where the transient action of the sinapism does not suffice, and where we hesitate before employing the blister. We have then no other recourse than frictions with tartar emetic or croton oil, and applications of thapsia. But these means are attended by inconveniences so serious, that we often hesitate to advise them. Tartar emetic produces an eruption which heals slowly and leaves indelible traces; hence, it has been generally given up. Croton oil and thapsia occasion intolerable itching, lasting a long time, and besides, often a painful swelling and a general eruption, and their action is slow to produce. If still employed, it is for want of better. Burgundy pitch is almost useless.

What is required to answer the purpose is an agent whose action is at once rapid and prolonged, and which provokes a sharp revulsion, without pain or itching. Does this agent exist? It did not formerly, or at least its properties were scarcely known, and it was not employed; but it certainly will be in the future. This agent is *pimento*, or rather its extract, which has just been made known by M. Lardy. It unites in effect, in the highest degree, the diverse conditions which we have enumerated. It acts with great rapidity, ten to twenty minutes, according to point of application and delicacy of the skin. Its action from the first is manifested by heat, slight smarting and redness. These phenomena increase for about three hours, then remain stationary, and the revulsive action continues as long as desired. However, after twenty-four hours in adults, and eight or ten hours in children, it is best to remove the plaster, or put another in a new place if further revulsion is desired. The heat and smarting caused are not painful, and do not hinder the patient from his occupation. There is no itching, and the action always remains localized. It may well be compared to that of mustard arrived at the half of its power, and thus remaining for twenty-four hours.

It may be seen from this brief exposition, what part may be drawn from this new revulsive in cases where a rapid and prolonged derivation is necessary; acute or chronic inflammation of the bronchia or throat, congestions of various organs, rheumatic pains, neuralgia, etc.

The color of the extract is beautiful red, identical with that of the dried fruit; when properly incorporated with some plastic mass, and spread on squares of paper, its application is easy. It is not necessary to heat it, as it adheres readily. When the parts are subjected to movements, it may be held in place by a bandage. Its action is augmented by pressure. After removal, if desired, the heat and smarting may be relieved immediately by application of starch. Care should be used not to get it in contact with the eyes, lips, or nose, on account of the smarting caused by it in those regions.

The author is sure that all who try the new revulsive will be satisfied with it, as he has been.

THERAPEUTICS OF TETANUS.—(*Dublin Med. Press.*) Anonymous. — *Chloroform* has had an extensive trial; it has been administered in large quantities, sometimes with apparent success. Simpson narcotised a child for thirteen consecutive days, using ℥ 100 with mercury. But the general result is that while all the fatal symptoms disappear on the inhalation of chloroform, they return on its removal with unabated violence, and the disease generally lands them to its fatal conclusion without delay. *Chloral hydrate* has now taken the place of chloroform in the treatment of tetanus, but without more success. There appears to be great tolerance of the drug, and a case is quoted of a child of $12\frac{1}{2}$ years who took more than 100gr. a day. Dr. Ballantyne, of Dalkeith, gave ℥ iij, in twenty-four hours, and ℥ vj. in five weeks, with success, the patient during this time being easily aroused to speak. It seems, however, to be a valuable drug in alleviating the symptoms. Its injection into the veins and its subcutaneous injection have not been so successful. *Calabar bean*, which, like chloral, affects the spinal cord, and has little or no action on the motor and sensory nerves, has been recently much employed. As with other drugs, its administration has been at one time apparently successful, and at another a perfect failure. It has, moreover, to be given in comparatively large doses. The spasms are controlled and the body heat sinks, and if the drug be withheld the paroxysms return, while if it be pressed the patient comes into a somewhat dangerous condition. A large dose is required to produce by subcutaneous injection contraction of the pupil, sometimes as much as $\frac{1}{3}$gr. every two hours. There is not much to be said in favor of either *opium* alone, or opium combined with chloral; while *nitrite of amyl*, *bromide of potassium*, and *conium* have been alike tried in vain. A more avorable report is given of *aconite*, the exhibition of which has been attended in some cases with remakable results. It lowered the pulse, which fell in one case from 135 to 60, with a simultaneous decrease of the convulsions; but the effects of the drug constitute in themselves a new danger which must be carefully controlled. Tendency to syncope, wakefulness, vertigo, dilatation and insensibility of the pupil; small, intermittent, and irregular pulse, and increased irritability of the nervous system are often the result of giving this remedy. The writer of the article referred to, believes that such a summary as he has given makes an appeal to pathology to throw fresh light upon this disease, and he hopes that some combination of these agents will be able to accomplish what each one of them singly has been found unable to accomplish.

We have no doubt that we shall one day find a remedy that is as really successful in the treatment of tetanus as the bromide of potassium has been found to be in some forms of epilepsy; but just as we are not indebted to pathology for the discovery of the therapeutic virtues of the bromide in epilepsy, so we are far from being sanguine that pathology will point out by-and-by the drug or combination of drugs which will cure the disease under consideration. In all probability the chemist or the botanist has already provided the remedy; and perhaps it remains for empirical experiment, rather than for physiology or pathology, to find it out.

